# Anti-citrullinated Protein Antibody Generation, Pathogenesis, Clinical Application, and Prospects

**DOI:** 10.3389/fmed.2021.802934

**Published:** 2022-01-12

**Authors:** Jiaxi Liu, Jinfang Gao, Zewen Wu, Liangyu Mi, Na Li, Yajing Wang, Xinyue Peng, Ke Xu, Fengping Wu, Liyun Zhang

**Affiliations:** Third Hospital of Shanxi Medical University, Shanxi Bethune Hospital, Tongji Shanxi Hospital, Shanxi Academy of Medical Sciences, Taiyuan, China

**Keywords:** rheumatoid arthritis, citrullination, ACPA, autoimmunity, autoantibody

## Abstract

Anti-citrullinated protein antibodies (ACPAs) are autoantibodies commonly observed in patients with rheumatoid arthritis (RA). Currently, most of the mechanisms of ACPA formation and bone destruction are well-understood, however, some unknown mechanisms still exist. There have been many new advances in ACPA-related clinical applications and targeted therapies. However, the existence of different ACPA subtypes is a limitation of targeted therapy. Herein, we present an overview of the process of ACPA generation, the underlying pathogenesis, and relevant clinical application and prospects.

## Introduction

Rheumatoid arthritis (RA) is a chronic erosive disease that can lead to joint deformity and loss of function. Anti-citrullinated protein antibodies (ACPAs) are autoantibodies against citrullinated peptides and proteins. The specificity of anti-cyclic citrullinated peptides (anti-CCP), which belong to ACPAs, is 88–98%. Therefore, ACPAs may be reliable markers for the early diagnosis of RA (1). Furthermore, they can predict the possibility of bone erosion in RA patients since ACPAs are mainly seen in RA, while citrullination is seen in many diseases (2). The citrullination process, how ACPAs damage joints, and if ACPAs can be used as therapeutic targets for RA, remain to be elucidated. This review will describe the process of ACPA generation, discuss the pathophysiological mechanisms underlying ACPAs action, and summarize the research progress in ACPA-based diagnosis, disease evaluation, and targeted therapy.

## ACPA Generation

### Protein Citrullination

Citrullination is mediated by peptidyl-arginine deiminase (PAD), which converts arginine to citrulline. It is a physiological post translational modification involved in brain development, apoptosis, epidermal differentiation, and chromatin regulation. In some cases, including RA, multiple sclerosis, and Alzheimer's disease, citrullination can be over represented (3). Proteins that are usually citrullinated include type II collagen, fibrinogen, α-enolase, filaggrin, histones, and vimentin (4). Citrullinated proteins are primarily produced by two subtypes of PADs, PAD2 and PAD4. PAD2 and PAD4 are usually found in neutrophils and monocytes (5). Physiologically, the function of PADs is limited (such as oxidizing environment present extracellular or transient nanomolar changes in intracellular calcium), and redundant citrullinated proteins are degraded. However, membranolytic damage caused by host perforin (in the joint) and bacterial pore-forming proteins (in extra-articular sites like gut and lungs) can induce PADs activation and hypercitrullination. These processes finally cause the superfluous production of ACPAs (6).

Calcium ions (Ca^2+^) are essential for PAD activation, but the intracellular calcium concentration in normal cells is much lower than that required for PAD activation. High Ca^2+^ concentration can occur locally in cells or in extreme conditions like apoptosis or necrosis (3). PADs are activated during cell death when PADs leak out of cells into the extracellular matrix or extracellular Ca^2+^ enters into cells (7). Accumulated citrullinated proteins have increased immunogenicity and are converted into autoantigens (8). Additionally, not all citrullination processes trigger ACPAs production. ACPAs are more likely to be produced in the presence of external environmental stimuli or autoimmune dysfunction (9).

### The Emergence of ACPAs

ACPAs are produced against various citrullinated protein antigens, including fibrinogen, vimentin, type II collagen, α-enolase, filaggrin, and histone (10). Smoking, silica exposure, and some air pollutants can lead to molecular changes in the lung and bronchoalveolar lavage fluid, resulting in increased expression of citrullinated proteins and/or peptides. Neutrophil extracellular traps (NETs) are extracellular fiber networks mainly composed of neutrophil DNA, which allow neutrophils to kill extracellular pathogens and minimize host cell damage (11). In patients with RA, NETs contain citrullinated vimentin and α-enolase, which can also stimulate autoantibody production (12).

**Figure 1:**
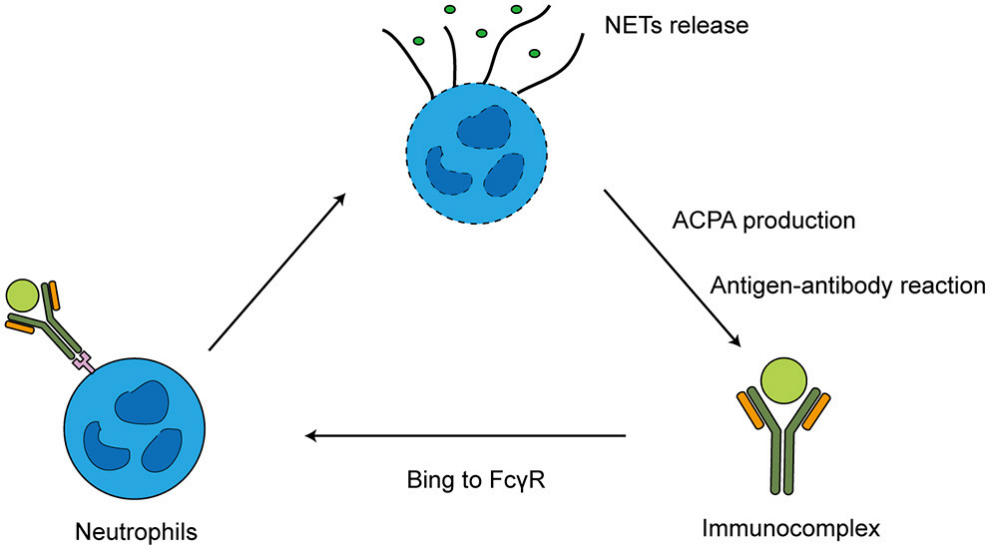
Interaction of ACPA and neutrophils.

Neutrophils and ACPAs interact through self-continuation. Excessive NETs formation can contribute to the production of deiminated antigens including citH2A and citH2B histones. When NETs release a large amount of citrullinated antigens to drive ACPA production, the immune complexes containing ACPAs can form more NETs. Then, the immune complexes interact with the FCγ receptor (FCγR) on other neutrophils, releasing degrading enzymes and reactive oxygen species, ACPAs and citrullination promote programmed cell death, leading to autoantigen release (13–15) (Figure 1). In RA patients' peripheral blood, ACPAs stimulate neutrophils to release PAD enzymes. Upon PAD4 activation, locally released citrullinated histones enhance the generation of highly mutated clonal B cells resulting in the generation of ACPAs (16).

**Figure 2:**
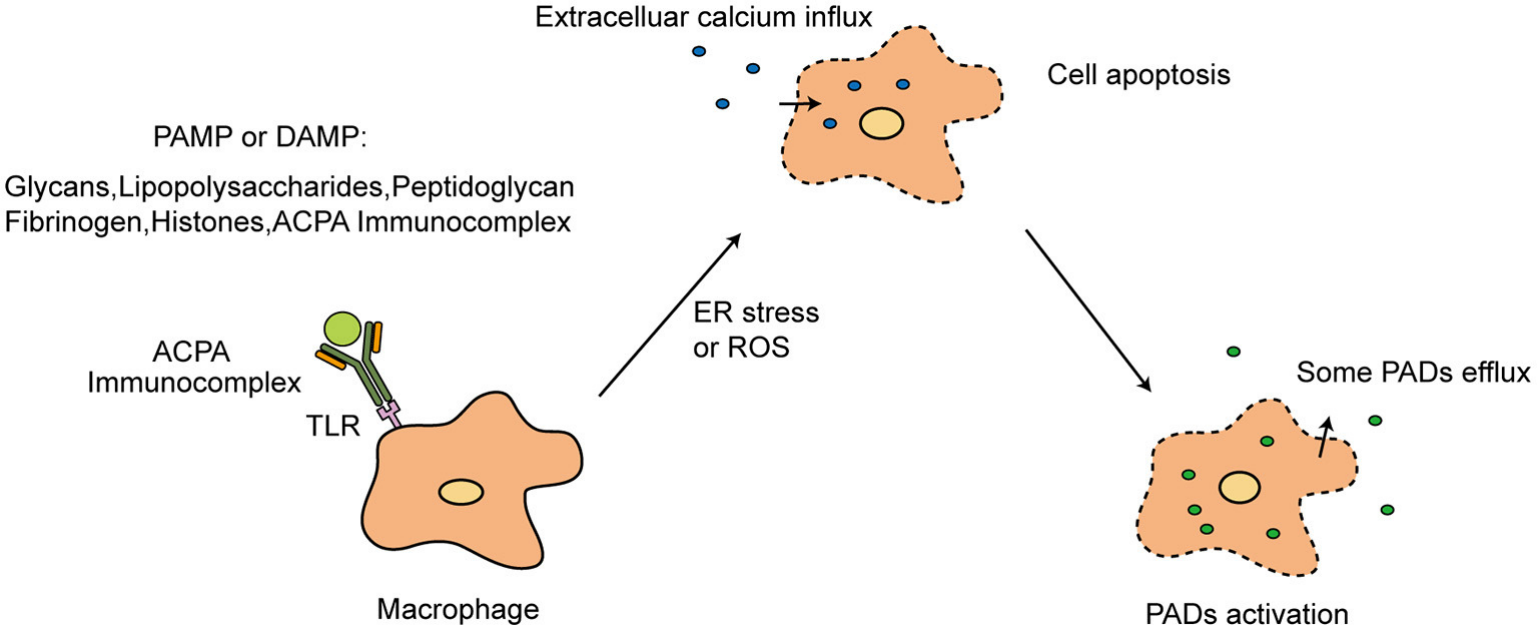
PAMP or DAMP in PADs activation. TLR, toll-like receptors; PAMP, pathogen associated molecular pattern; DAMP, Damage-associated molecular patterns; ER, Endoplasmic reticulum; ROS, reactive oxygen species.

Various noxious agents have a potential to activate toll-like receptors, which belong to pattern recognition receptors (expressed mainly by cells of the innate immune system, such as macrophages). Then, the triggered innate immune response activate Ca^2+^-mediated PAD of granulocytes and macrophages (17). Furthermore, Damage-associated molecular patterns (DAMP) have been shown to induce NETs production through pattern recognition receptors (18). Citrullinated histones and their immune complexes have been reported to function as DAMP in RA. The cell apoptosis induced by pathogen associated molecular pattern (PAMP) and DAMP can also lead to an unlimited influx of Ca^2+^ (Figure 2) (19–22). Then, those Ca^2+^ activate PADs.

In the presence of a susceptible major histocompatibility complex (MHC), different citrullinated proteins appear in different MHC molecular backgrounds to activate T cells. They promote B cell maturation and activation, leading to the consecutive production of ACPAs. Immunoglobulin G (IgG) is the primary type of ACPA (23). Different types of autoantigens and antibodies make ACPAs specific, and they appear in different individuals before the onset of the disease, carrying out systemic diffusion and epitope spreading. This process leads to a mix of ACPAs and an increase in the ACPA titer (24, 25), making targeted therapy incredibly difficult.

When using CCP to detect ACPAs, almost all ACPAs react. Because of non-cyclic citrullinated peptides, the term ACPA is broader than anti-CCP antibody (26). Anti-CCP antibodies are able to recognize citrullinated fibrinogen and citrullinated myelin basic protein. Moreover, antibodies specific for these two antigens are largely crossreactive, but the crossreactivity is incomplete (27, 28).

RA patients' sera react diversely with the different citrullinated peptides. 29.08% of sera react with vimentin, 37.59% with alpha-enolase, 31.21% with fibrin, 29.97% with type II collagen and 28.37% with filaggrin. These citrullinated peptides have also been found to have high specificity for RA. Moreover, RA Patients' sera with various reactivities to one or more citrullinated peptides do not present difference in disease severity (29). In a study of pre-clinical RA cases, the prevalence of ACPA subtypes varies. Histone 2A is 5.5%. Histone 2B is 8.6%. Vimentin is 14.9%. Fibrinogen is 17.7%. Biglycan is 4.7%. In addition, two or more ACPA subtypes were present in 15.7% of pre-RA cases (30). These researches proves that ACPAs are heterogeneous and react differently to different citrullinated proteins.

## ACPAs and Bone Destruction in RA

### The ACPA Immune Response

The ACPA immune response can be divided into two stages, namely, the “first hit” and the “second hit.” The serum ACPA titer increases, and the immune response begins before the onset of RA. The initial break of tolerance that leads to the production of low level and low activity ACPA (“the first hit”) is often caused by environmental changes or some genetic background that is linked to RA susceptibility. It can last for many years without causing any symptoms (31). After some additional arthritogenic triggers, citrulline-specific B cells may receive T cell help, inducing an inflammatory autoimmune response (“the second hit”) (32). Then, ACPA levels increase, antibody diversity expands, and the ACPA response matures (13).

Furthermore, HLA-DRB1 encode shared epitope that is highly associated with the development of ACPA-positive RA. It includes DRB1^*^0401, DRB1^*^0409, DRB1^*^ 0404, and DRB1^*^0101. Young-onset RA (≤40 years old) is often associated with DRB1^*^0401 and DRB1^*^0404, while late-onset RA (≥60 years old) is associated with the presence of DRB1^*^0101. Patients with shared epitopes usually have increased levels of HLA-DR on B cells, which interact with T cell receptors (17). The HLA-DRB1^*^13 alleles have a protective effect on the transition from ACPA-positive autoimmunity to ACPA-positive RA and are associated with lower ACPA levels (33).

In the process of antigen presentation, HLA shared epitopes are presented to T cells, which promote B cell proliferation induced by citrullinated peptides. Then, the ACPA reactions expand, resulting in the “second hit,” in which T cells promote the expression of ACPAs in B cells. A wide range of somatic high-frequency mutations, epitope spreading, antibody titer increase, class conversion, and recombination occur. The increase in the ACPA serum titer, the broadening of the antigen recognition pattern, and the glycosylation of the ACPA Fc segment all provide evidence. T cells assist B cells in expressing ACPAs and trigger the expansion of the ACPA response before arthritis onset (34).

### ACPA Pathogenicity and Induction of B Cell Immune Tolerance

During the ACPA immune response, glycosylation can affect the stability and biological activity of antibodies (35). Glycosylation can be observed in the variable regions of the heavy and light chains (31). In general, 15–25% of IgG antibodies contain Fab glycan, whereas over 90% of ACPA-IgG molecules carry Fab glycan (36). ACPA-IgG can also be glycosylated in the tail of Fc and this is related to their pathogenicity. ACPA variable region glycosylation is related to B-cell tolerance and survival. The acquisition of variable domain glycans could enable ACPA-expressing B cells to breach tolerance (31). ACPA-IgG are highly and extensively glycosylated in the variable domain. In addition, the relationship between variable region polysaccharides and the development of ACPAs requires further study (34).

Almost all (>90%) ACPA-IgG molecules contain N-glycans in their variable regions. However, only a few variable N-glycosylation sites (defined by the common sequence asn-x-ser) encoded by region genes can accommodate this glycan. In contrast to ACPA-IgG, ACPA-IgM does not show enhanced glycosylation in the variable region. T cells are essential for the selection and expansion of ACPA-expressing B cells, possibly by facilitating the introduction of N-glycosylation sites in the ACPA-IgG variable region. This T cell–B cell interaction also mediates the increased usage of isotypes and epitope spreading observed before disease onset. Acquisition of N-glycans in the ACPA variable domain is a process that requires repeated T cell–dependent B cell hypermutation events, potentially as a result of multiple hits that occur with varying kinetics (37). The introduction of ACPA Fab glycans is likely the result of somatic hypermutation during maturation of the ACPA response. The introduction of N-glycans in the variable domain is dependent on T cell help inside or outside the germinal center. However, the role of Fab glycan in the function of ACPA-IgG remains unclear (32).

The N-glycosylation sites are not randomly accumulated. They are the result of a non-random process, which indicates that ACPAs can obtain a survival advantage because of these polysaccharides. Furthermore, ACPA-specific B cells can circulate in the joint as memory B cells, which, then, undergo additional germinal center passage and/or differentiate into ACPA-secreting plasma cells (31). In addition to glycosylation, the level of ACPA sialylation also plays an important role in bone destruction. Reduced sialylation is a common IgG feature in patients with RA and in collagen-induced arthritis (CIA) mouse models. In CIA mice, blocking activated B cell sialylation at the gene level leads to exacerbation of arthritis. Artificial sialylated ACPAs can weaken the activity of ACPAs and reduce the damage of ACPAs to joints. Therefore, sialylation can be used as a potential target for the treatment of ACPA-positive RA (38).

### ACPA Pathogenesis

ACPAs can interact with osteocytes, chondrocytes, and immune cells in the articular cavity to initiate an inflammatory reaction (39). ACPAs and citrullinated protein can form immune complexes. These immune complexes combine with C1q and activate complement system through classical pathway (40). L.A. Trouw et al. (41) found that ACPAs activate the complement system *in vitro via* both classical and alternative pathways, and the extent of complement activation is positive correlated with anti-CCP antibody levels. What's more, the immune complexes induce the release of chemotactic factors C3a and C5a (40). In the ACPA-containing environment, the complement system play a vital role in inducing the pathogenesis of RA from the pre-clinical to clinical stage (42).

ACPAs can also directly or indirectly participate in bone destruction (39). Macrophages are activated by ACPAs through the formation of immune complexes, which then promote the production of pro-inflammatory cytokines and indirectly participate in bone destruction. Resting-state macrophages are usually located in synovial tissue. They are powerful immune effectors that can significantly promote the inflammatory response and joint destruction when stimulated (43, 44). Furthermore, they can selectively activate the extracellular signal-regulated kinase (ERK) and c-Jun N-terminal kinase (JNK) pathways through direct interaction between the Fab variable region of ACPAs and citrullinated glucose regulatory protein 78, leading to the activation of NF-κB and promotion of TNF-α production (45, 46).

**Figure 3:**
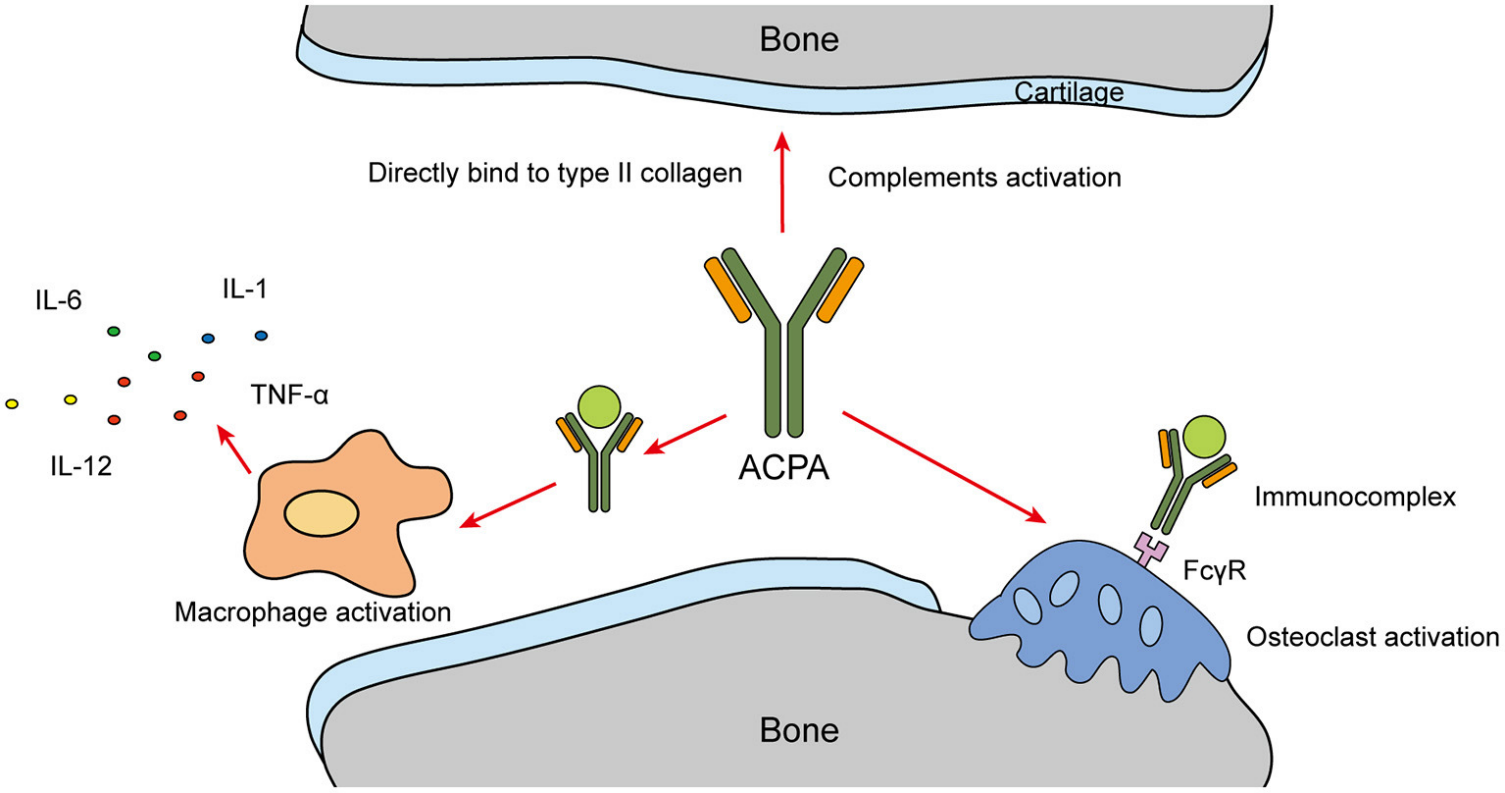
ACPA bone destruction.

ACPAs can also directly target osteoclasts (OCs). The immune complex formed by ACPAs and citrullinated peptide binds with FCγR, activates OCs and promotes proinflammatory cytokine production (39, 47). Immunocomplexes that contain antibodies with different degrees of glycosylation can change the differentiation and activation of OC precursors. The glycosylation of the immunoglobin Fc of ACPAs enhances their affinity for FCγR, and increases their ability to induce OC activation and bone erosion (48). In RA patients, ACPAs interacting with type II collagen can directly bind with cartilage components and antigens on the surface of articular cartilage (15). ACPAs cross-react with type II collagen and induce arthritis and structural damage by activating complements, leading to decreased proteoglycan levels and severe arthritis (49) (Figure 3).

## Clinical Application of ACPAs

### Diagnosis, Disease Prediction, and Assessment

ACPAs are mostly observed in patients with RA. As the specificity of ACPAs for RA was above 90%, it was included in the 2010 ACR/EULAR RA classification standard. The presence of ACPAs has a positive predictive value of 63% for RA progression within 1 year in individuals with recent (<1 year) arthralgia. Low levels of ACPAs can be detected in 1–3% of healthy individuals, and can exist for many years without causing obvious clinical symptoms in some people (34). Murata et al. (50) found that the ACPA titer level fluctuates in some patients. Moreover, a weak correlation between the ACPA titer level and disease activity was observed and fluctuation in the ACPA titer level could predict relapse in patients with RA remission. According to Anca Catrina's review, when individuals reach RA diagnosis, the ACPA repertoire remains relatively stable over time with small changes in ACPA levels and only occasional seroconversion (13).

In asymptomatic individuals, ACPAs are not sufficient to predict the development of RA, but the detection of ACPAs helps to determine the diagnosis, evaluate the prognosis of patients, and guide treatment decisions (31, 51). Serum ACPA levels are relatively stable in the course of the disease, while rheumatoid factor (RF) levels change. ACPA-positive B cells undergo multiple cycles of germinal center reaction, thus accumulating cell mutations and carrying out homotypic transformation. In contrast, RF-positive B cells undergo a limited germinal center response and are activated by innate immune mechanisms. The citrulline-specific immune response can produce long-lived plasma cells and a stable titer of ACPAs, while the RF response can produce short-lived plasma cells and fluctuating levels of RF (52) (Table 1). In addition, patients with strongly positive ACPAs and RF positivity in the absence of joint symptoms may have significant extra-articular involvement, including recurrent scleritis, pleuropericarditis, or interstitial lung disease (53). Moreover, high levels of ACPAs are associated with greater susceptibility to ocular diseases (54).

**Table 1:** Comparison of ACPA and RF characteristics.

	**ACPAs**	**RF**
Antibody type (15, 55)	Mainly IgG, sometimes IgA and IgM	Mainly IgM, sometimes IgA
**Disease relativity** (56)	Mostly in RA	In many autoimmune diseases
N- Glycosylation (57)	Extensive	Limited
**Germinal center response** (15)	Repetitive	Limited
Somatic hypermutation (58)	Extensive	Limited
**B cell activation** (15)	T cell-dependent	May or may not be T cell-dependent
Plasma cell production (52)	Long lived plasma cells	Short lived plasma cells
**Time of appearance** (59)	Before the onset of RA	Before the onset of RA

*ACPAs, anti-citrullinated protein antibodies; RF, rheumatoid factor; Ig, immunoglobulin; RA, rheumatoid arthritis*.

ACPA positivity is related to poor prognosis, including systemic extra-articular complications and joint damage. Compared to ACPA negative patients, ACPA positive patients are more likely to develop erosive disease, as demonstrated using radiography and ultrasonography, especially in the fifth metatarsophalangeal joints (60). ACPA-IgG levels are relatively stable and rarely turn negative in all phases of RA. The fluctuation of ACPA-IgG levels cannot reflect the activity of RA, nor can it predict the flare of the disease (34). However, in a longitudinal cohort study in RA patients diagnosed from 2000 onward, ACPA-positive RA was not more severe than ACPA-negative RA in terms of the patients' relevant outcomes, including physical functioning and restrictions at work (61).

### Efficacy Prediction

ACPA-positive and ACPA-negative RA have been shown to be related to different genetic backgrounds. HLA shared epitopes are more likely to induce ACPA-positive RA. HLA-DRB1^*^03 is more likely to cause ACPA-negative RA (62), which confirms that these two diseases have different pathophysiological mechanisms (63), such as higher disease severity and higher remission rates. Compared with the ACPA-negative group, the bone mass density and cortical thickness of the ACPA-positive group were significantly decreased, while the cortical pore area was significantly increased. The results of a systematic review of 10 studies on the role of ACPAs in predicting joint injury in RA in which 3,065 patients were included (average study time was 4.7 years) showed that ACPA positivity is an important predictor of joint erosion in patients with RA (2).

ACPA-positive RA and ACPA-negative RA respond differently to the traditional DMARD methotrexate, which can reduce the possibility of progression from undifferentiated arthritis to RA in ACPA-positive patients. However, there was no such reduction in ACPA-negative patients (62). In addition, glucocorticoids play a more significant role in disease amelioration in ACPA-positive RA, which indicates that ACPAs may serve as a marker to guide the treatment of early RA (64).

For RA patients with higher baseline ACPA titer, abatacept has better efficacy than the TNF inhibitor infliximab. During abatacept treatment, the inhibition of T cells was stronger in ACPA-positive patients than that in ACPA-negative patients. At 3 months, significant reductions in RF and ACPA titers were observed in the abatacept continuous treatment group. Furthermore, the decrease in the ACPA titer could be used as an independent predictor of abatacept persistence at 12 months because of sustained therapeutic response. Hence, when the disease activity is not under control or the titer of ACPAs continues to increase after 3 months' of abatacept use, RA patients should switch from abatacept to other DMARDs (65).

In RA, the reaction of ACPAs to citrullinated antigen is called “ACPA reactivity” (66). ACPA reactivity is considered to be related to bone destruction in RA. The ACPA reactivity level significantly decreases in the first 3 months of DMARD treatment, and then remains stable. Furthermore, the lower the ACPA reactivity, the easier it is to achieve methotrexate monotherapy remission in 6 months. After 12 and 24 months, radiologic progression is not significantly associated with baseline ACPA responsiveness (67).

In a Swedish clinical trial, serum samples from baseline and 3-month follow-up were available from 316 patients. DMARDs-naive RA patients with symptoms duration < 1 year were treated with methotrexate for 3 months, followed by randomization to add-on therapy with either sulfasalazine and hydroxychloroquine or infliximab in patients with a 28-joint disease activity score (DAS28) > 3.2 at the 3-month evaluation. The proportion of patients testing ACPA-positive declined significantly between 0 and 24 months regarding anti citrullinated peptides derived from vimentin, whereas anti-CCP antibody remained unaltered. Reversion from positive at baseline to negative at 2 years is significantly more common regarding anti citrullinated peptides derived from vimentin compared with anti-CCP antibodies. Moreover, anti citrullinated peptides derived from vimentin seroreversion remained associated with less occurrence of radiological progression (68).

### Periodontal Disease and ACPAs

Periodontal disease (PD) is a chronic inflammatory condition related to aberrant microbe that affect the supporting tissues around teeth. PD can destruct oral mineralized and non-mineralized connective tissues. Infection of Porphyromonas gingivalis, a periodontal pathogen, cause the activation of peptidylarginine deiminase (PPADs) that generates citrullinated proteins and triggers the synthesis of ACPAs. Then, the cross-reactivity of periodontal-generated APCAs to antigens present in the joint aggravate the inflammation associated with RA. Besides Porphyromonas gingivalis, Actinobacillus actinomycetemcomitansis is another periodontal pathogen. It can activate citrullination enzymes in host neutrophils through pore-forming toxin leukotoxin A. Exposure to leukotoxic Actinobacillus actinomycetemcomitans was confirmed in RA patients with PD and was positively associated with ACPA levels (69, 70).

ACPAs concentration is much more higher among RA patients with PD compared with RA patients or PD patients. A significant association was found between PD and RA, and the association was more evident in patients with more severe periodontal disease and higher RA disease activity. In addition, associations between cumulative IgG titer against periodontopathogens and ACPAs presence suggest a synergistic effect of periodontopathogens to ACPAs development (71–73). Although Non-Surgical Periodontal Treatment has limited effects to reduce the RA clinical scores, the periodontal status of RA patients receiving DMARDs is better than the untreated RA patients, and the suppression of TNF-α to treat RA may be beneficial in PD treatment (69).

## Targeted ACPA Therapy

### Anti-citrullination Treatment

At present, some bioactive compounds have been found to irreversibly target PAD. The earliest effective bioactive compounds were F-amide and Cl-amidine, which irreversibly inhibit PAD4 and citrullination by modifying the active site of cysteine (74). Cl-amidine has been shown to reduce disease severity in CIA mice (75). However, F-amide and Cl-amidine are still in the stage of animal trials, and their clinical efficacy needs further evaluation.

Since the PAD4 gene is a susceptibility gene for RA, it can be used as a therapeutic target. It is known that PAD4 can affect the disease severity of CIA mice and enhance collagen-induced inflammatory responses. After PAD4 gene knockout, the degree of arthritis in CIA mice significantly decreased, but disease onset was not prevented. PAD4 inhibitors are good candidates for drug development because their use is not associated with physiological abnormalities. A novel PAD4-selective inhibitor, GSK199, could reduce clinical disease activity and joint destruction in CIA mice. However, it did not markedly reduce citrullination or the number of ACPAs in joints (76, 77).

### Vaccines Against Citrullinated Proteins

Various vaccines have been found effective in arthritis treatment in animal models. Anti-cytokine vaccine IL23-K1 produce great amounts of anti-IL23 antibodies in mice. It is also highly protective against joint destruction and inflammation. Vacacine RTFP-2, a RANKL-TNF-like core fusion protein, produce a total inhibition of osteoclastogenesis *in vitro*. PADRE-BAFF vaccine yield high titers of neutralizing B-cell activating factor (BAFF) antibodies and ameliorate arthritis in rats. Vaccine CEL-2000, constructed from a peptide of Type II collagen, limit the progression of arthritis in CIA mice. Most importantly, the development of “Rheumavax” has been evaluated with promising results in a phase I RA clinical trial. Rheumavax is also the first and only trial existing in humans (78).

Rheumavax is a novel vaccine for the treatment of RA. It comprises dendritic cells modified by NF-κB inhibitors exposed to four citrullinated peptide antigens. Its efficacy has been demonstrated in animal models. The NF-κB subunit is overexpressed in RA synovial tissues. Dendritic cells lacking NF-κB subunits can inhibit the existing immune response *in vivo* in an antigen-specific manner by inducing a suppressive CD4^+^ regulatory T (Treg) cell population. One month after injection, the proportion of Treg cells increased, and the proportion of effector T cells decreased. Inflammatory cytokines and disease activity also decreased in patients receiving treatment (79, 80). In an animal model of RA, inoculation with multi-epitope peptides derived from citrullinated autoantigen promoted Treg cell production and inhibited Th17 cell function, inducing immune tolerance and reducing joint inflammation (81). Vaccine therapy may be used in high-risk groups with multiple patients with RA in the family. However, due to the diversity of citrullinated proteins, whether the vaccines developed for certain peptides can be used in all high-risk groups needs further study.

### Immunotherapy

Since ACPAs are secreted by effector B cells, B cell treatment is expected to significantly improve the symptoms of ACPA-positive patients. When rituximab is used to treat RA, the ACPA levels significantly decrease. However, these levels usually do not turn negative. In many RA patients, a relatively stable citrullinated protein-related response exists, which may be maintained by plasma cells residing in the bone marrow, spleen, and synovial tissue. In a 2-year period of B-cell depletion, autoantibodies had a significantly shorter life span than that of physiologically protective antibodies. Hence, there might be a therapeutic window for therapies that target plasma cells (34, 82, 83).

Auto-reactive B cells, which are considered to play a key role in many autoimmune diseases, can produce ACPAs. Auto-reactive B cells can be used as targets for the treatment of ACPA-positive RA. A synthetic CCP antigen suitable for combination with B cell receptors has been synthesized. After reduction by nitroreductase, the activated CCP antigens, which are in close proximity to the B cell surface, selectively bind to B cell receptors and initiate cell death. Furthermore, the binding of ACPAs and citrullinated peptide was inhibited by introducing a carboxyl-p-nitrobenzyl (CNBz) blocking group into the side chain of the citrulline residue. However, this method has some limitations. The self-reactive B cells in patients with RA are polyclonal. Therefore, the curative effects are uncertain. Moreover, these findings are based on animal studies (84).

### Targeting ACPAs

ACPAs are important in the process of bone destruction in RA. Targeting ACPAs is expected to alleviate bone destruction and the clinical symptoms of RA. Research shows that two kinds of chains originating from the fibrinogen α chain can destroy the binding of ACPAs with CCP (85). The therapeutic anti-citrullinated protein antibodies synthesized on this basis specifically bind citrulline at position three of histone 2A and histone 4. The antibodies showed strong anti-inflammatory activity in CIA mice. Therapeutic ACPAs inhibit murine and human NET formation and bind to NETs *in vitro* and *in vivo*, potentially initiating clearance by macrophages (86). Furthermore, citrulline epitopes of human fibrinogen can be grafted into natural stable peptide scaffolds, and then peptide binders can be used to directly target and neutralize ACPAs. The peptide scavenger binds ACPAs with high affinity, specificity, and stability (87). This method, however, needs to be further studied in the human body.

## Discussion and Outlook

Many studies have been conducted on ACPA generation and pathogenesis. Accumulated citrullinated proteins trigger ACPA production and different types of ACPAs appear depending on the different antigens and antibody isotypes. ACPA pathogenesis can be divided into “two hits.” After the “second hit,” ACPA levels markedly increase. In the ACPA immune response, glycosylation can affect the stability and biological activity of antibodies, enhancing ACPA pathogenicity. Moreover, ACPAs directly or indirectly participate in bone destruction.

There are many new advances in the clinical application and targeted therapy of ACPAs. Based on the characteristics of ACPAs, many new ideas in diagnosis, curative effect prediction, and related vaccines are created. However, the ACPA subtypes differ depending on the different antigens and antibody isotypes. Targeted therapy can only target some of them, which is a limitation of targeted therapy. According to Sieghart et al. (23), ACPA-IgG has a higher sensitivity (57.9%) in RA diagnosis than ACPA-IgA (34.1%) and ACPA-IgM (28.6%). However, Chirivi et al. (86) found that therapeutic ACPAs can inhibit NET formation and become a potential therapy for inflammatory arthritis. Therefore, the effect of ACPAs remains controversial. With more exploration of ACPAs and clinical trials, new breakthroughs will be made in the generation, pathophysiology, clinical diagnosis, and treatment of ACPA-positive RA.

## Author Contributions

JL and JG performed and wrote the manuscript. JL, LM, NL, and XP collected the references and designed the table. JL and ZW drew the figures. KX, YW, and FW modified the manuscript. LZ designed the manuscript and approved the final manuscript for publication. All authors read and approved the final manuscript.

## Funding

This work was supported by the National Natural Science Foundation of China (Grant Number 81771768) and the applied basic research project of Shanxi Science and Technology Department (Grant Number 201901D111416).

## Conflict of Interest

The authors declare that the research was conducted in the absence of any commercial or financial relationships that could be construed as a potential conflict of interest.

## Publisher's Note

All claims expressed in this article are solely those of the authors and do not necessarily represent those of their affiliated organizations, or those of the publisher, the editors and the reviewers. Any product that may be evaluated in this article, or claim that may be made by its manufacturer, is not guaranteed or endorsed by the publisher.

## Abbreviations

ACPAs: anti-citrullinated protein antibodies
RA: rheumatoid arthritis
anti-CCP: anti-cyclic citrullinated peptide
PAD: peptidyl arginine deiminase
NETs: neutrophil extracellular traps
FCγR: Fc gamma receptor
CCP: cyclic citrullinated peptide
CIA: collagen-induced arthritis
ERK: extracellular signal regulated kinase
JNK: c-Jun N-terminal kinase
RF: rheumatoid factor
DMARDs: disease-modifying antirheumatic drugs
PAD4: peptidyl arginine deiminase type 4
CNBz: carboxyl-p-nitrobenzyl.
